# Effects of Selected Anionic **β**-Cyclodextrins on Persistence of Blood Glucose Lowering by Insulin Glargine after Subcutaneous Injection to Rats

**DOI:** 10.1155/2011/195146

**Published:** 2011-12-11

**Authors:** Keiko Uehata, Takayuki Anno, Kayoko Hayashida, Keiichi Motoyama, Taishi Higashi, Fumitoshi Hirayama, Naomi Ono, James D. Pipkin, Kaneto Uekama, Hidetoshi Arima

**Affiliations:** ^1^Graduate School of Pharmaceutical Sciences, Kumamoto University, 5-1 Oe-honmachi, Kumamoto 862-0973, Japan; ^2^Faculty of Pharmaceutical Sciences, Sojo University, 4-22-1 Ikeda, Kumamoto 860-0082, Japan; ^3^CyDex Pharmaceuticals, Inc., Lenexa, KS 66214-1643, USA

## Abstract

Insulin glargine is a synthetic long-acting insulin product used for patients with diabetes mellitus. In this study, to obtain the further desirable blood-glucose lowering profile of insulin glargine, we investigated the effects of **β**-cyclodextrin sulfate (Sul-**β**-CyD) and sulfobutylether **β**-cyclodextrin (SBE7-**β**-CyD) on physicochemical properties of insulin glargine and pharmacokinetics/pharmacodynamics of insulin glargine after subcutaneous injection to rats. Sul-**β**-CyD and SBE7-**β**-CyD increased solubility of insulin glargine. SBE7-**β**-CyD suppressed the formation of oligomer and enhanced the dissolution rate of insulin glargine from its precipitate, compared to that of Sul-**β**-CyD. Additionally, we revealed that after subcutaneous administration of an insulin glargine solution, SBE7-**β**-CyD, but not Sul-**β**-CyD, increased bioavailability and sustained the blood-glucose lowering effect, possibly due to the inhibitory effects of SBE7-**β**-CyD on the enzymatic degradation at the injection site. These results suggest that SBE7-**β**-CyD could be a useful excipient for sustained release of insulin glargine.

## 1. Introduction

Diabetes is a rapidly growing health problem worldwide and chronic disease wherein the pancreas does not produce enough insulin (type 1 diabetes), or the body does not respond correctly to insulin and relative insulin deficiency (type 2 diabetes). It can be a life-threatening disease and can also lead to serious complications such as cardiovascular disease, kidney failure, blindness, and nerve damage [1–3]. According to the World Health Organization, the number of people living with diabetes is estimated to increase from 172 million in 2000 to 366 million in 2030 [4]. The global diabetes epidemic has devastating effects on not only patients and their families but also national economies.

**Figure 1:**
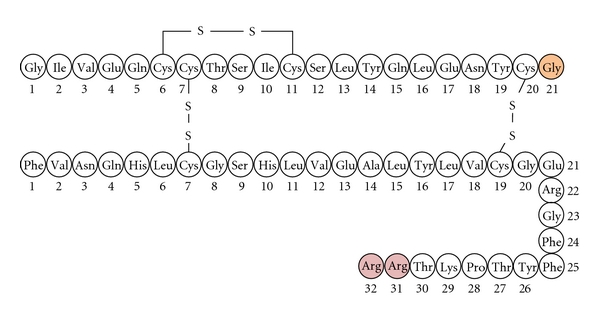
Amino acid sequence and location of intermolecular disulfide bonds of insulin glargine.

Human insulin is a major backbone for the treatment of diabetes. Although human insulin has contributed much in clinical treatment of diabetes for a long time, there are still some difficulties and challenges of hypoglycemia and short half-life. In order to overcome these drawbacks, insulin glargine (Lantus), an insulin analogue (C_267_H_404_N_72_O_78_S_6_, MW = 6,063) was developed by replacing asparagine at the position of 21 of the A chain with glycine, and two arginines were added to the C-terminus of the B chain in human insulin (Figure 1). It has a prolonged duration of action after subcutaneous injection and, therefore, can provide a basal insulin level for 24 hours by once daily injection [5]. This alteration results in low aqueous solubility at neutral pH [6]. Insulin glargine is supplied in an acidic solution, which becomes neutralized at the injection site, leading to the formation of microprecipitates from which insulin glargine is slowly released into the circulation [6].

Cyclodextrins (CyDs) are known to form inclusion complexes with various guest molecules [7, 8]. However, the low aqueous solubility of natural CyDs, especially *β*-CyD, has restricted their range of applications. To improve their solubility, alkylated, hydroxyl alkylated, sulfated, sulfobutyl alkylated, and branched CyDs have been developed [9–12]. Of these hydrophilic CyDs, maltosyl-*β*-CyD (G_2_-*β*-CyD), 2-hydroxypropyl-*β*-CyD (HP-*β*-CyD), *β*-CyD sulfate (Sul-*β*-CyD) and sulfobutyl ether-*β*-CyD (SBE-*β*-CyD) have higher solubility in water and relatively low hemolytic activity and thus have potential as pharmaceutical excipients for parenteral preparation [8, 9, 13]. In fact, natural *β*-CyD has a toxic effect on kidney, which is the main organ for the removal of CyDs from the systemic circulation and for concentrating CyDs in the proximal convoluted tubule after glomerular filtration [14]. Actually, amorphous mixtures of highly water-soluble *β*-CyDs such as HP-*β*-CyD and SBE-*β*-CyD have very low systemic toxicity, compared with *β*-CyD.

We previously reported the effects of hydrophilic *β*-CyDs on the aggregation of bovine insulin in aqueous solution and its adsorption onto hydrophilic surfaces [15–17]. Of the CyDs tested, G_2_-*β*-CyD potently inhibited insulin aggregation in a neutral solution and its adsorption onto the surfaces of glass and polypropylene tubes. In addition, SBE-*β*-CyDs showed different effects on insulin aggregation in phosphate buffer (pH 6.8, *I* = 0.2), depending on the degree of substitution (DS) of the sulfobutyl ether group, SBE4-*β*-CyD (DS = 3.9) showed deceleration of insulin aggregation, and SBE7-*β*-CyD (DS = 6.2) showed acceleration [17]. Furthermore, we reported that after subcutaneous administration of insulin solution to rats, SBE4-*β*-CyD rapidly increased plasma insulin level and maintained higher plasma insulin levels for at least 8 h, possibly due to the inhibitory effects of SBE4-*β*-CyD on the enzymatic degradation and/or the adsorption of insulin onto the subcutaneous tissue at the injection site [18]. More recently, we have demonstrated that SBE4-*β*-CyD enhanced both bioavailability and prolonged the blood-glucose lowering effect of insulin glargine after subcutaneous administration to rats, probably due to the inhibitory effects of interaction with SBE4-*β*-CyD on the enzymatic degradation at the injection site [19]. However, it is still unknown whether other anionic *β*-CyD derivatives such as Sul-*β*-CyD and SBE7-*β*-CyD show the improved bioavailability and sustained-glucose lowering effects for insulin glargine. Therefore, the objective in the present study is to evaluate the potential use of anionic *β*-CyD derivatives, such as Sul-*β*-CyD and SBE7-*β*-CyD, on not only bioavailability of insulin glargine but also the sustained-blood-glucose lowering effects. In addition, the effects of Sul-*β*-CyD and SBE7-*β*-CyD on physicochemical properties and pharmacokinetics/pharmacodynamics of insulin glargine were examined.

## 2. Materials and Methods

### 2.1. Materials

Insulin glargine was supplied by Sanofi-Aventis (Paris, France). SBE7-*β*-CyD was provided by CyDex (Kansas, USA). Sul-*β*-CyD with an average degree of substitution of 10.7 was prepared by a nonregional selective method as described previously [20]. Recombinant trypsin (EC 3.4.21.4) of proteomics grade was purchased from Roche Diagnostics (Tokyo, Japan). Phosphate buffer (pH 9.5, *I* = 0.2) was prepared according to the US Pharmacopeia; 0.1 mol/L phosphoric acid solution and 0.1 mol/L sodium hydroxide solution were mixed, followed by the addition of sodium chloride. All other materials were of analytical reagent grade, and deionized double-distilled water was used.

### 2.2. Spectroscopic Studies

Fluorescence and circular dichroism (CD) spectra were measured at 25°C using a HITACHI fluorescence spectrophotometer F-2500 (Tokyo, Japan) and a JASCO J-720 polarimeter (Tokyo, Japan), respectively.

### 2.3. Solubility Studies

Excess amounts of insulin glargine were shaken in phosphate buffer (pH 9.5, *I* = 0.2) in the absence and presence of the selected anionic *β*-CyDs at 25°C. After equilibrium was attained, the solutions were filtered with Millex GV filter 0.22 *μ*m, and the insulin glargine dissolved was determined by high-performance liquid chromatography (HPLC) with Agilent 1100 series (Tokyo, Japan) under the following conditions: Merck Superspher 100 RP-18 column (4 *μ*m, 3 mm × 250 mm, Tokyo, Japan), a mobile phase of phosphate buffer (pH 2.5) and acetonitrile and a gradient flow, increasing the ratio of the acetonitrile (25–40%) over 30 min, a flow rate of 0.55 mL/min, a detection of UV at 214 nm.

### 2.4. Ultrafiltration Studies

Ultrafiltration studies were performed using stirred ultrafiltration cells model 8010 (Millipore, Tokyo, Japan) applied with YM30 ultrafiltration discs (MWCO = 30,000) in phosphate buffer (pH 9.5, *I* = 0.2) in the absence and presence of the selected anionic *β*-CyDs at 25°C under nitrogen current. Insulin glargine levels in filtrates were determined by HPLC as described above.

### 2.5. Particle Size Determination

Particle sizes of insulin glargine (0.1 mM) with or without the selected anionic *β*-CyDs (10 mM) in phosphate buffer (pH 9.5, *I* = 0.2) were measured by Zetasizer Nano (Malvern Instruments, Worcestershire, UK).

### 2.6. Dissolution Study of Insulin Glargine

Insulin glargine (0.1 mM) dissolved in phosphate buffer (pH 9.5, *I* = 0.2) in the absence and presence of the selected anionic *β*-CyDs (10 mM) was precipitated by a pH shift to 7.4. After centrifugation (2,500 rpm, 10 min), the supernatant was discarded, and then phosphate buffer (pH 7.4, *I* = 0.2) was newly added to the precipitate at 25°C. At appropriate intervals, an aliquot of the dissolution medium was withdrawn, centrifuged at 2,500 rpm for 10 min, and analyzed for the insulin glargine by HPLC as described above.

### 2.7. Stability of Insulin Glargine against Tryptic Cleavage

Insulin glargine (0.1 mM) in phosphate buffer (pH 9.5, *I* = 0.2) was incubated with recombinant trypsin (0.02 mg/mL) in the absence and presence of the selected anionic *β*-CyDs at 37°C. At appropriate intervals, 5 *μ*L of sample solution was withdrawn and determined intact insulin glargine level by HPLC. The rate constants (*k*_*c*_) and stability constants (*K*_*c*_) of 1 : 1 complexes of insulin glargine/*β*-CyDs under the tryptic cleavage were determined by a quantitative analysis according to the following equation, where *k*_0_ and [*CyD*]_*t*_ stand for the rate constants without CyD and the total concentration of CyD, respectively [21]:

(1) $$\frac{{\left[CyD\right]}_{t}}{k_{0}-k_{\text{obs}}}=\frac{1}{k_{0}-k_{c}}\cdot {\left[CyD\right]}_{t}+\frac{1}{K_{c}\cdot \left(k_{0}-k_{c}\right)}.$$

### 2.8. Pharmacokinetics and Pharmacodynamics of Insulin Glargine

The solution (0.582 mL/kg) containing insulin glargine (2 IU/kg) in phosphate buffer (pH 9.5, *I* = 0.2) in the absence and presence of the selected anionic of *β*-CyDs was subcutaneously injected in male Wistar rats (200–250 g), and, at appropriate intervals, blood samples were taken from the jugular veins. Serum insulin glargine and glucose were determined by Glyzyme Insulin-EIA Test Wako (Wako Pure Chemicals, Osaka, Japan) and Glucose-CII-Test Wako (Wako Pure Chemicals Ind., Osaka, Japan), respectively. Serum glucose levels after the administration of a solution of insulin glargine with or without the selected anionic *β*-CyDs were expressed as a percentage of the initial glucose level before injection.

### 2.9. Statistical Analysis

Data are given as the mean ± S.E.M. Statistical significance of means for the studies was determined by analysis of variance followed by Scheffe's test. *P*-values for significance were set at 0.05.

## 3. Results and Discussion

### 3.1. Spectroscopic Studies

**Figure 2:**
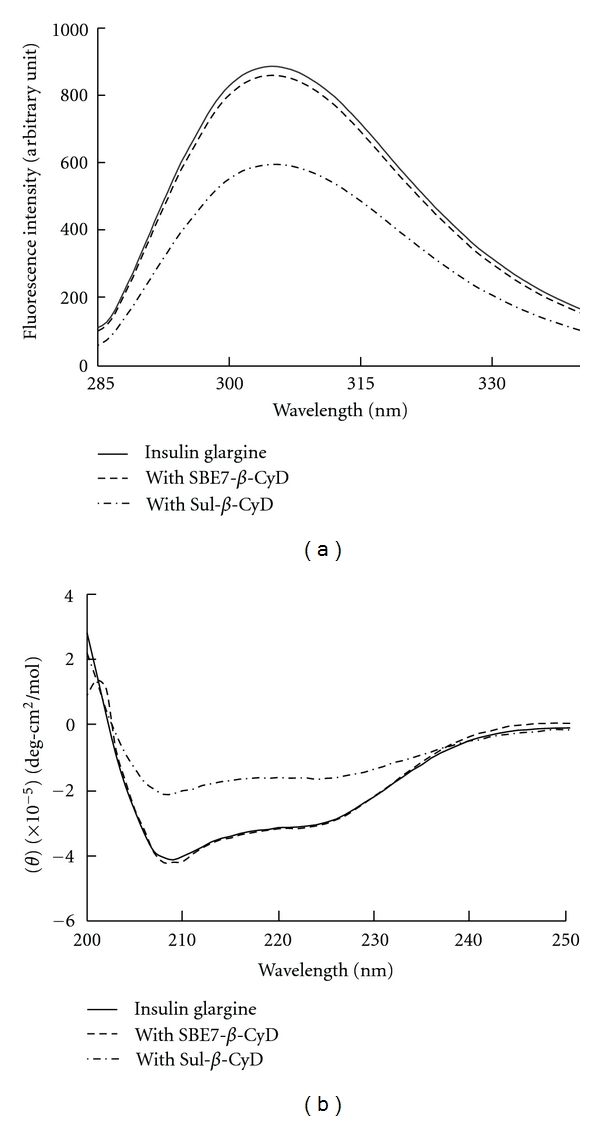
Effects of Sul-*β*-CyD and SBE7-*β*-CyD (10 mM) on fluorescence spectrum (a), circular dichroism spectrum of insulin glargine (0.1 mM) in phosphate buffer (pH 9.5, *I* = 0.2) at 25°C. The excitation wavelength in measurement of fluorescence spectrum was 277 nm.

CyDs have been claimed to interact with hydrophobic residues exposed on protein surfaces and thereby to decrease the aggregation of proteins [22, 23]. We previously reported that SBE4-*β*-CyD inhibited the aggregation of bovine insulin in neutral solution, possibly due to the interaction of SBE4-*β*-CyD with aromatic side chain of insulin such as B26-tyrosine, A19-tyrosine, B1-phenylalanine, and B25-phenylalanine [17]. Also, our recent study has shown that SBE4-*β*-CyD increased solubility of insulin glargine, enhanced the dissolution rate from its precipitate, and inhibited its aggregation in phosphate buffer (pH 9.5, *I* = 0.2), with all possibly due to the formation of complex with insulin glargine [19]. In the present study, to reveal whether the selected anionic CyD derivatives, Sul-*β*-CyD, and SBE7-*β*-CyD, interact with insulin glargine, the effects of both of the selected anionic *β*-CyDs (10 mM) on the fluorescence and CD spectra of insulin glargine were investigated (0.1 mM) (Figure 2). To obtain the clear solution of insulin glargine (0.1 mM) in spectroscopic studies, insulin glargine with the selected anionic *β*-CyDs was dissolved in phosphate buffer (pH 9.5, *I* = 0.2) at 25°C. The fluorescence intensity of tyrosine of insulin glargine at 306 nm was remarkably quenched by the addition of Sul-*β*-CyD (10 mM) while SBE7-*β*-CyD (10 mM) quenched slightly (Figure 2(a)). As tyrosine is a hydrophobic amino acid having a phenyl group in the molecule, these selected anionic *β*-CyDs may interact with those aromatic amino acid residues of insulin glargine. The apparent 1 : 1 stability constants (*K*_*c*_) of the insulin glargine/Sul-*β*-CyD complex and insulin glargine/SBE7-*β*-CyD complex were determined by the titration curves of the fluorescence intensity against the concentration of the selected anionic *β*-CyD with the Scott's equation [21]. The *K*_*c*_ values of insulin glargine/Sul-*β*-CyD complex and insulin glargine/SBE7-*β*-CyD complex in phosphate buffer (pH 9.5, *I* = 0.2) at 25°C were calculated to be 14 ± 3 M^−1^ and 18 ± 4 M^−1^, respectively. The CD spectrum of insulin glargine (0.1 mM) showed negative bands at 210 nm and 220 nm in phosphate buffer (pH 9.5, *I* = 0.2) (Figure 2(b)). The two negative bands assigned to *α*-helics (a characteristic feature of the monomer) and *β*-sheets (a predominant feature of dimer) structures [24]. In the presence of Sul-*β*-CyD (10 mM), the both negative bands at 210 nm and 220 nm in the CD spectrum of insulin glargine remarkably increased. These results indicate that Sul-*β*-CyD decreased the content of monomer and dimer of insulin glargine in phosphate buffer (pH 9.5, *I* = 0.2). Meanwhile, the CD spectrum of insulin glargine in the presence of SBE7-*β*-CyD was changed only very slightly, compared to that of insulin glargine alone, suggesting that SBE7-*β*-CyD did not induce a conformational change of insulin glargine in phosphate buffer (pH 9.5, *I* = 0.2). To gain insight into the mechanism of the interaction mode of these anionic *β*-CyDs with insulin glargine, further investigation should be required using NMR technique. Collectively, these results strongly suggest that the interaction mode of Sul-*β*-CyD and SBE7-*β*-CyD against insulin glargine is much different; namely, Sul-*β*-CyD, but not SBE7-*β*-CyD, induces topological change of insulin glargine in phosphate buffer (pH 9.5, *I* = 0.2), and this difference may contribute to explaining the observed differences in* in vivo* behavior as well.

### 3.2. Solubility Studies

**Figure 3:**
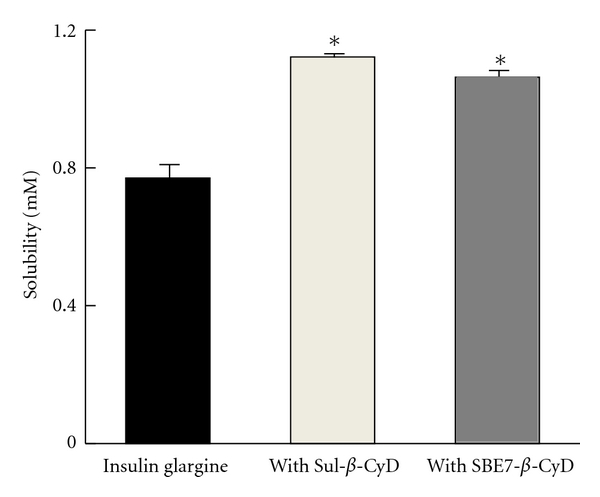
Effects of Sul-*β*-CyD and SBE7-*β*-CyD (10 mM) on solubility of insulin glargine in phosphate buffer (pH 9.5, *I* = 0.2) at 25°C. Each value represents the mean ± S.E.M. of 3 experiments. *P < 0.05, compared to insulin glargine.

The preferred presentation for administration by subcutaneous injection is a clear aqueous solution, and so this is the desired form for administration of insulin and its analogues. However, insulin or insulin analogues are poorly soluble in aqueous solution, in particular at around their isoelectric point (pI), approximately pH 6.7, close to the physiological pH [25]. Hence, the effects of Sul-*β*-CyD and SBE7-*β*-CyD on solubility of insulin glargine were examined. As shown in Figure 3, the solubility of insulin glargine in phosphate buffer at pH 9.5 was significantly increased by the addition of Sul-*β*-CyD or SBE7-*β*-CyD and so appears to be due to an inclusion complexation and electrostatic interaction between insulin glargine and the selected anionic *β*-CyDs. These results suggest that Sul-*β*-CyD and SBE7-*β*-CyD potentially enhance the solubility of insulin glargine in phosphate buffer.

### 3.3. Ultrafiltration Studies

**Figure 4:**
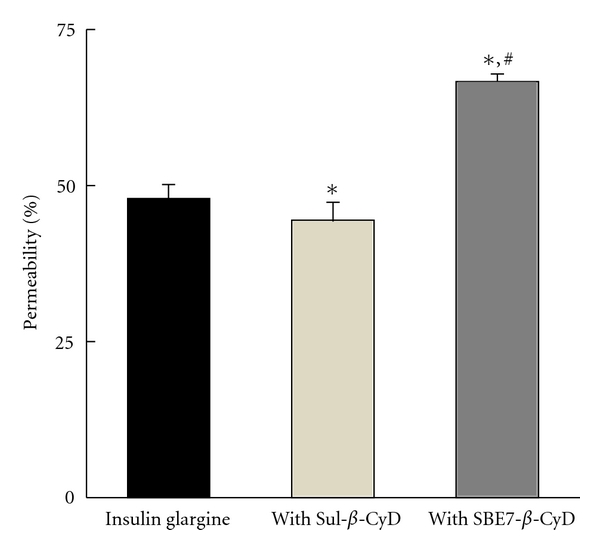
Effects of Sul-*β*-CyD and SBE7-*β*-CyD (10 mM) on permeation of insulin glargine (0.1 mM) through ultrafiltration membrane having nominal molecular weight limit of 30,000 in phosphate buffer (pH 9.5, *I* = 0.2) at 25°C. Each value represents the mean ± S.E.M. of 17 and 5 experiments for insulin glargine and with Sul-*β*-CyD or SBE7-*β*-CyD, respectively. *P < 0.05, compared to insulin glargine. ^#^ *P* < 0.05, compared to Sul-*β*-CyD.

The aggregation and self-association of insulin and its analogue are elicited by many kinds of factors such as the concentration of insulin, pH, temperature, shaking, and so on [5, 6]. Insulin glargine forms dimer, tetramer, hexamer, and further soluble oligomers by noncovalent interactions such as proceeding from self-association [26, 27]. Therefore, we performed ultrafiltration studies to estimate the effects of Sul-*β*-CyD and SBE7-*β*-CyD on self-association of insulin glargine using the membrane YM30 (MWCO = 30,000) in phosphate buffer (pH 9.5, *I* = 0.2). As shown in Figure 4, insulin glargine in the absence of *β*-CyDs permeated the ultrafiltration membrane by approximately 50%. SBE7-*β*-CyD significantly enhanced the permeation of insulin glargine up to almost 70%. These results suggest that interaction with SBE7-*β*-CyD results in dissociation of such soluble oligomers of insulin glargine. On the other hand, the presence of Sul-*β*-CyD slightly, but significantly decreased the permeation of insulin glargine to approximately 45%, although not accompanied by observable formation of insoluble aggregates of insulin glargine under the prevailing experimental condition. Recall from above, that Sul-*β*-CyD decreased the contents of monomer and dimer of insulin glargine in phosphate buffer (pH 9.5, *I* = 0.2) (Figure 2(b)). Therefore, these results, taken together, indicate that Sul-*β*-CyD enhanced the association of soluble oligomer of insulin glargine from its monomer and dimer.

### 3.4. Particle Size Determination

The apparent particle sizes of insulin glargine were determined by the dynamic light scattering method in the absence and presence of Sul-*β*-CyD and SBE7-*β*-CyD (Table 1). Particle size of insulin glargine alone in phosphate buffer (pH 9.5, *I* = 0.2) was determined as 744 ± 82 nm. Particle sizes of insulin glargine in the presence of Sul-*β*-CyD and SBE7-*β*-CyD increased significantly to 1334 ± 164 nm and 1575 ± 228 nm, respectively. It is estimated that the sulfate and sulfobutyl groups of Sul-*β*-CyD and SBE7-*β*-CyD are both strongly hydrated in aqueous solution. Therefore, these results suggest that Sul-*β*-CyD and SBE7-*β*-CyD enhanced the particle size of insulin glargine in phosphate buffer.

### 3.5. Dissolution Study of Insulin Glargine

**Figure 5:**
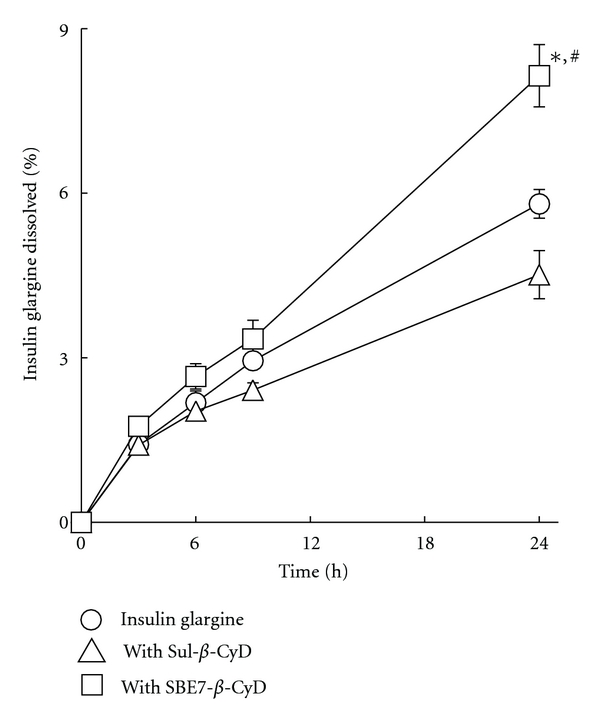
Effects of Sul-*β*-CyD and SBE7-*β*-CyD (10 mM) on dissolution from isoelectric precipitation of insulin glargine in phosphate buffer (pH 9.5, *I* = 0.2) at 25°C. The initial concentration of insulin glargine was 0.1 mM and then precipitated in phosphate buffer (pH 7.4). Each point represents the mean ± S.E.M. of 3 experiments. *P < 0.05, compared to insulin glargine. ^#^ *P* < 0.05, compared to Sul-*β*-CyD.

Insulin glargine is believed to precipitate at the physiological pH after subcutaneous injection of the solution due to pI (about pH 6.7), which is followed by a sustained release of insulin glargine over 24 h at an injection site because of its extremely low solubility in aqueous solution at pH of around pI [6]. In order to investigate the effects of Sul-*β*-CyD and SBE7-*β*-CyD on the sustained release of insulin glargine, the dissolution rate of insulin glargine from isoelectric precipitates formed with or without *β*-CyDs was determined (Figure 5). Insulin glargine (0.1 mM) was dissolved in phosphate buffer (pH 9.5) in the presence and absence of *β*-CyDs (10 mM), and then isoelectric precipitation of insulin glargine was obtained after pH shift from 9.5 to 7.4. Then, the release rate of insulin glargine was determined in phosphate buffer (pH 7.4) in the absence of selected anionic *β*-CyDs. SBE7-*β*-CyD significantly increased the dissolution rate of insulin glargine after 24 h, compared to insulin glargine alone. This enhancing effect of SBE7-*β*-CyD on the dissolution rate is consistent with its solubilizing effect as shown in Figure 3. On the other hand, Sul-*β*-CyD appeared to decrease the dissolution rate of insulin glargine after 24 h; however, no statistical significance was found. The inhibitory effect of Sul-*β*-CyD on the dissolution rate of insulin glargine from its precipitate may be ascribed to the enhancement of the association of insulin glargine molecules that is dominant over the solubilizing effect of Sul-*β*-CyD on insulin glargine. To reiterate, SBE7-*β*-CyD, and not Sul-*β*-CyD, increases dissolution of insulin glargine from its precipitate.

### 3.6. Stability of Insulin Glargine against Tryptic Cleavage

**Figure 6:**
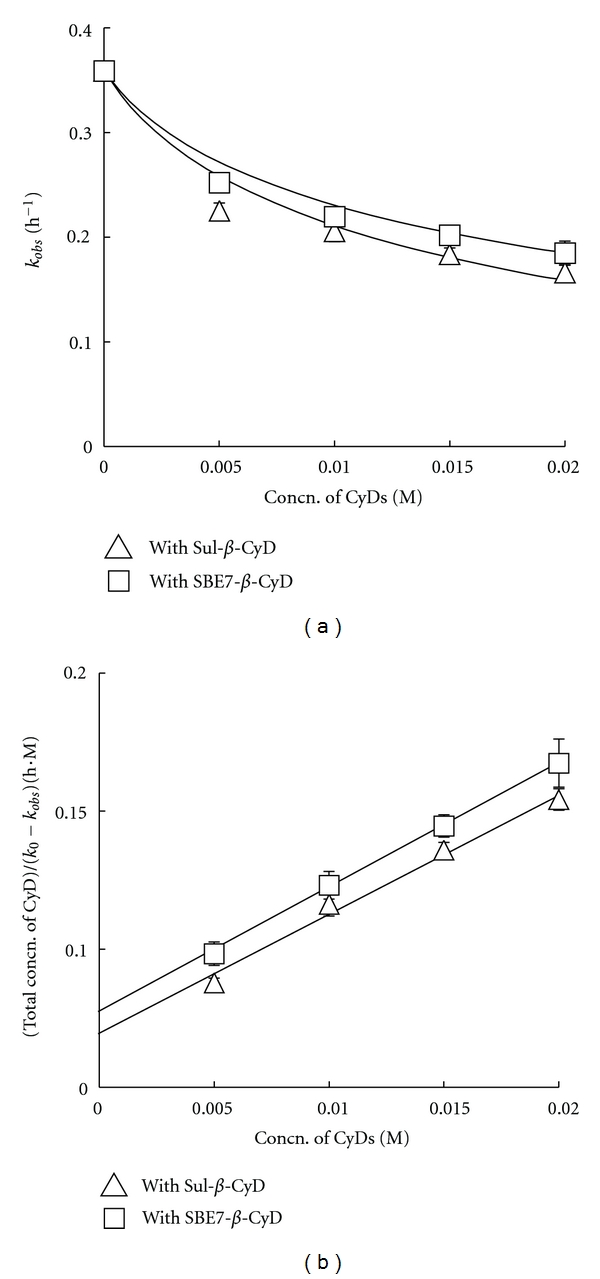
Effects of Sul-*β*-CyD and SBE7-*β*-CyD (5 to 20 mM) on tryptic cleavage (2 IU) of insulin glargine (0.1 mM) in phosphate buffer (pH 9.5, *I* = 0.2) at 37°C. Each point represents the mean ± S.E.M. of 3 experiments.

Insulin and its analogues are digested by proteases such as trypsin, which cleaves insulin at the carboxyl side of residues B22-arginine and B29-lysine, at an injection site and systemic circulation [28]. Therefore, a resistance towards enzymatic degradation is required for a formulation of insulin or its analogues to demonstrate improvement in bioavailability. Next, the effects of Sul-*β*-CyD and SBE7-*β*-CyD on stability of insulin glargine against trypsin digestion were investigated. In this study, insulin glargine was digested by trypsin at 2 IU at pH 9.5 at 37°C with different degradation rates in the absence and presence of *β*-CyDs. As shown in Figure 6(a), the apparent degradation rate constant of insulin glargine alone (*k*_0_) was 0.357 ± 0.004 h^−1^. Meanwhile, the apparent rate constants (*k*_obs_) in the presence of Sul-*β*-CyD and SBE7-*β*-CyD decreased with the increase in the concentration of these *β*-CyDs. The decline in the *k*_obs_ value in the SBE7-*β*-CyD system was more than that in the Sul-*β*-CyD system. The rate constants (*k*_*c*_) and stability constants (*K*_*c*_) of the 1 : 1 complex calculated with the regression lines shown in the Figure 6(b) were 0.129 ± 0.009 h^−1^ and 244 ± 24 M^−1^ in the Sul-*β*-CyD system and 0.137 ± 0.014 h^−1^ and 182 ± 22 M^−1^ in the SBE7-*β*-CyD system, respectively. These results suggest that the inhibition of tryptic cleavage of insulin glargine by Sul-*β*-CyD and SBE7-*β*-CyD may be ascribed to the formation of the soluble oligomer and soluble complex with insulin glargine (Figures 3 and 4), respectively, resulting from decreasing the extent of the free insulin glargine that could be easily digested by trypsin. Our previous studies revealed that the *k*_*c*_ and *K*_*c*_ values in the SBE4-*β*-CyD system were 0.145 ± 0.012 h^−1^ and 144 ± 18 M^−1^, respectively [19]. Therefore, it is evident that the inhibitory effect of SBE7-*β*-CyD on enzymatic degradation of insulin glargine is more potent than that of SBE4-*β*-CyD.

Recently, it has been reported that the aspartic acid residue existing in the catalytic pocket of trypsin is responsible for attracting and stabilizing positively charged lysine and/or arginine on the substrate peptide [29]. Therefore, the insulin glargine/Sul-*β*-CyD interaction or insulin glargine/SBE7-*β*-CyD complex is speculated to ameliorate the interaction between the negatively charged aspartic acid in the catalytic pocket of trypsin and positively charged lysine and/or arginines mentioned earlier, since Sul-*β*-CyD and SBE7-*β*-CyD have negative charge originating from the sulfate and sulfonate groups, respectively. This hypothesis in which the insulin glargine/Sul-*β*-CyD interaction and insulin glargine/SBE7-*β*-CyD complex ameliorate the interaction between the aspartic acid and lysine and/or arginines is supported by the finding that the aromatic amino acid residues in insulin glargine which are capable of interacting with *β*-CyDs (at B24-, B25-phenylalanines, B26-tyrosine, and B28-proline) locate near the three digestive sites by trypsin (B22-B23, B29-B30, and B31-B32) [17]. These results suggest that Sul-*β*-CyD and SBE7-*β*-CyD act as stabilizers of insulin glargine against enzymatic degradation by their respective interactions with insulin glargine.

### 3.7. Subcutaneous Administration of Insulin Glargine/*β*-CyDs Solutions to Rats

**Figure 7:**
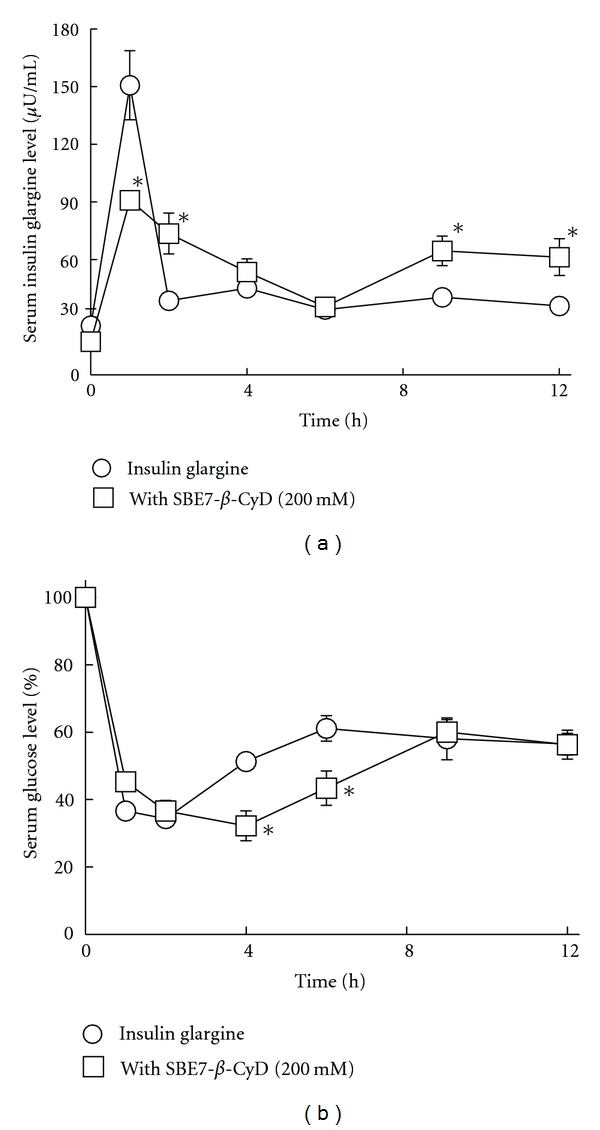
Effects of SBE7-*β*-CyD (200 mM) on serum insulin glargine (a) and glucose (b) levels after subcutaneous administration of insulin glargine (2 IU/kg) to rats. Each point represents the mean ± S.E.M. of 4–6 experiments. *P < 0.05, compared to insulin glargine.

To confirm whether Sul-*β*-CyD and SBE7-*β*-CyD are useful excipients for insulin glargine *in vivo*, we evaluated the effects of the *β*-CyDs on pharmacokinetics and pharmacodynamics of insulin glargine after subcutaneous injection to rats. In our preliminary studies, we found that neither Sul-*β*-CyD (100 mM) nor SBE7-*β*-CyD (100 mM) changed the serum glucose level-time profiles remarkably in comparison with that of insulin glargine alone (2 IU/kg) after subcutaneous injection to rats (data not shown). Taking the positive results of SBE7-*β*-CyD in ultrafiltration (Figure 2) and dissolution (Figure 3) studies by contrast to those of Sul-*β*-CyD into account, further *in vivo* investigation was performed with a higher concentration of SBE7-*β*-CyD.  Figure 7(a) and Table 2 show the serum insulin glargine level-time profiles and pharmacokinetics parameters, respectively, after subcutaneous administration of insulin glargine (2 IU/kg) with or without SBE7-*β*-CyD (200 mM) in phosphate buffer (pH 9.5) to rats. When insulin glargine alone was injected, the maximum level (*C*_max⁡_) of insulin glargine and the time (*T*_max⁡_) required to the reach *C*_max⁡_ after injection were 150 *μ*U/mL and 1.00 h, respectively. In the presence of SBE7-*β*-CyD (200 mM), *C*_max⁡_ significantly decreased to 91.60 *μ*U/mL although *T*_max⁡_ did not change remarkably, compared to that of insulin glargine alone. The area under the serum insulin glargine level-time curve (AUC) in the SBE7-*β*-CyD system (200 mM) up to 12 h (687.86 (*μ*U/mL)·h) was significantly increased, compared to those of insulin glargine alone (582.99 (*μ*U/mL)·h). In addition, SBE7-*β*-CyD (200 mM) extended the mean residence time (MRT) of the serum insulin glargine level significantly, comparing with that of insulin glargine alone. These results indicate that SBE7-*β*-CyD sustained the serum insulin glargine level.

Figure 7(b) and Table 3 show the serum glucose level-time profiles and pharmacodynamics parameters after subcutaneous administration of insulin glargine (2 IU/kg) with or without SBE7-*β*-CyD (200 mM) in the phosphate buffer (pH 9.5) to rats. When insulin glargine alone was administered, the time to nadir blood glucose concentration (*T*_nadir_) was 1.6 h after injection, and then the blood glucose levels recovered within 6 h to basal level. On the other hand, insulin glargine administered with SBE7-*β*-CyD significantly retained the blood-glucose lowering effect up to 6 h after administration. *T*_nadir_ was significantly increased in the insulin glargine/SBE7-*β*-CyD system. Further, the insulin glargine/SBE7-*β*-CyD system showed the tendency to augment the area under serum glucose level-time curve (AUC_G_). The retained blood-glucose lowering effects and enhancement of *T*_nadir_ by the addition of SBE7-*β*-CyD may be contributed to (1) the inhibitory effects of SBE7-*β*-CyD on the enzymatic degradation of insulin glargine (Figure 6) and (2) the enhancement of solubility and the dissolution rate of insulin glargine by SBE7-*β*-CyD (Figures 3–5). However, the enhancement of bioavailability and persistence of the blood-glucose lowering effect of insulin glargine after subcutaneous injection to rats by SBE7-*β*-CyD was not superior to that of SBE4-*β*-CyD. The reason for this may be due to the difference in adsorption of insulin glargine onto the subcutaneous tissue at injection site between the SBE7-*β*-CyD and SBE4-*β*-CyD systems [19]. To gain insight into the detailed mechanism, further study on the adsorption of insulin glargine in the presence of SBE-*β*-CyDs onto subcutaneous tissue at injection site is underway. These results suggest that SBE7-*β*-CyD increased the bioavailability and persistence of the blood-glucose lowering effect of insulin glargine after subcutaneous administration of an insulin glargine solution to rats.

## 4. Conclusions

In the present study, we revealed that Sul-*β*-CyD and SBE7-*β*-CyD increased solubility of insulin glargine. Furthermore, SBE7-*β*-CyD suppressed the formation of oligomer and enhanced the dissolution rate of insulin glargine from its precipitate, compared to that of Sul-*β*-CyD. In addition, we demonstrated that SBE7-*β*-CyD increased the bioavailability and persistence of the blood-glucose lowering effect of insulin glargine after subcutaneous administration of an insulin glargine solution to rats, probably due to the inhibitory effects of SBE7-*β*-CyD on the enzymatic degradation at the injection site, resulting from the interaction with insulin glargine molecules. These findings indicate that SBE7-*β*-CyD can be a useful excipient for a peakless profile of insulin glargine.

## Figures and Tables

**Table 1:** Particle size of insulin glargine with or without Sul-*β*-CyD and SBE7-*β*-CyD (10 mM) in phosphate buffer (pH 9.5). The particle size was measured by a Zetasizer Nano. The concentrations of insulin glargine and *β*-CyDs were 0.1 mM and 10 mM, respectively. Each value represents the mean ± S.E.M. of 5–7 experiments.

System	Diameter (nm)
Insulin glargine	744 ± 82
With Sul-*β*-CyD	1334 ± 164*
With SBE7-*β*-CyD	1575 ± 228*

Uehata et al. [19]

**Table 2:** *In vivo* pharmacokinetics parameters of insulin glargine with or without SBE7-*β*-CyD (200 mM). (1) Time required to reach the maximum serum insulin glargine level. (2) Maximum serum insulin glargine level. (3) Area under the serum insulin glargine level-time curve up to 12 h after-administration. (4) Mean residence time in plasma. Each value represents the mean ± S.E.M. of 4–6 experiments. *P < 0.05, compared to insulin glargine.

System	T_max⁡_^(1)^ (h)	C_max⁡_^(2)^ (*μ*U/mL)	AUC^(3)^ ((mU/mL)h)	MRT^(4)^ (h)
Insulin glargine	1.00 ± 0.00	150.00 ± 17.90	582.99 ± 30.27	1.83 ± 0.08
Insulin glargine/SBE7-*β*-CyD	1.40 ± 0.24	91.60 ± 3.04*	687.86 ± 20.57*	2.12 ± 0.04*

**Table 3:** *In vivo* pharmacodynamics parameters of insulin glargine with or without SBE7-*β*-CyD (200 mM). (1) Time to nadir blood glucose concentration. (2) Nadir blood glucose concentration. (3) The cumulative percentage of change in serum glucose levels up to 12 h after-administration. (4) Mean residence time in plasma. Each value represents the mean ± S.E.M. of 5-6 experiments. *P < 0.05, compared to insulin glargine.

System	T_nadir_^(1)^ (h)	C_nadir_^(2)^ (%)	AUC_G_^(3)^(% · h)	MRT_G_^(4)^ (h)
Insulin glargine	1.60 ± 0.16	33.14 ± 1.10	544.66 ± 31.73	2.28 ± 0.03
Insulin glargine/SBE7-*β*-CyD	3.50 ± 0.05*	32.05 ± 4.73	612.36 ± 40.84	2.29 ± 0.01
